# Supplementary material for: Gut microbiota differences between paired intestinal wall and digesta samples in three small species of fish
Source: PeerJ. 2022 Feb 22;10:e12992. doi: 10.7717/peerj.12992 (PMC8877339; doi:10.7717/peerj.12992)
Supplement: Table S2 [file peerj-10-12992-s002.docx]

**PowerSoil Extraction Protocol**

**Before starting**

• Set the heat block at 65 oC

**Preservative removal**

If the samples are stored in a preservative, first remove it.

1. Intestines and solid faeces in ethanol:
   - Clean plates with bleach and ethanol, let it dry.
   - Transfer the intestine to the plate and let it dry.
   - Using a sterile razor, cut the intestine.
   - Transfer to the corresponding PowerBead tube.
2. Soft or dissolved faeces in ethanol:
   - Centrifuge at 13,000g for 2 minutes.
   - Discard the supernatant as much as possible and let it dry.
   - Transfer the pellet to the PowerBead tube with the help of a tip.
   - Add bead solution to the tube and pipette up and down resuspending the

     pellet.
   - Transfer the solution to the corresponding PowerBead tube.

**Extraction protocol**

1. Add **60 ul** of **C1** solution to PowerBead tube and **vortex** thoroughly (15 sec)
2. **Heat the tubes for 15 min at 65 oC** in the heat block. **Meanwhile:**
   • Add 250 ul C2 solution to empty 1.5 ml tubes and label them. • Add 200 ul C3 solution to empty 1.5 ml tubes and label them. • Label 1xN 2 ml tubes.
    • Label 2xN Collection Tubes (provided).

   • Label 1xN Spin Filters (provided).
3. **Tissuelyse** the tubes **10 min at freq 20** on a Qiagen TissueLyser.
4. **Centrifuge** the tubes **13,000xg for 3 min.**
   **[TIP]**: If >12 samples, first centrifuge half of the samples, and the other half while performing the next step (8+8, 12+12, etc.). That way resuspension of the pellet is avoided. Do the same in the following centrifuge steps.
5. **Transfer the supernatant** (500-600 ul) to the 1.5 ml tubes **containing C2** solution and **vortex** briefly (5 sec).

   **[TIP]:** Better use P200 pipette and transfer the supernatant in 2-3 steps.
6. Incubate for **10 min at 4 oC** (fridge).
7. **Centrifuge** the tubes **13,000xg for 1 min.**
8. **Transfer the supernatant** (600-700 ul) to the 1.5 ml tubes **containing C3** solution and **vortex** briefly (5 sec).

**[WARNING]:** Avoid transferring the pellet! Better transfer less volume.

1. Incubate for **10 min at 4 oC** (fridge).

**[MEANWHILE]:** Shake the C4 solution and add 1200 ul (600+600) to 2 ml tubes.

1. **Centrifuge** the 1.5 ml tubes **13,000xg for 1 min.**
2. **Transfer the supernatant** (up to 750 ul) to the 2 ml tubes **containing C4** solution and **vortex** briefly (5 sec).

**[WARNING]:** Avoid transferring the pellet! Better transfer less volume.

1. Load **650 ul onto a Spin Filter**, **centrifuge 8,000xg for 1 min** and discard Collection Tube and **place the Spin Filter in a clean Collection Tube.**
2. **Repeat** the previous step 2 more times.
    **[TIP]:** You can skip the previous steps using the Vacuum Manifold.
3. **Add 500 ul of C5** solution and centrifuge at **13,000xg for 1 min**. Check if the liquid spun through.

   **[WARNING]:** Avoid splashing C5 solution onto the Spin Filter! Keep the pipette tip

   above the C5 solution bottle until one drop has fallen.
4. **Place the Spin Filter in a clean Collection Tube** and centrifuge again at **13,000xg for 1 min**.
5. **Place the Spin Filter in a clean Collection Tube** and **add 50 ul EB** or TET buffer to the **center of the Spin Filter**.
6. Incubate **15 min at 37-40^o^C.**
   **[MEANWHILE]:** Label 5xN LoBind 1.5 ml tubes with definitive names.

   **[TIP]:** Better make aliquots to avoid contamination in the following steps.
7. Centrifuge **13,000xg for 1 min** .
8. Discard the Spin Filter and **transfer the extract to 1.5 low bind tubes.**
