# Supplementary figures and images for: Gut microbiota differences between paired intestinal wall and digesta samples in three small species of fish
Source: PeerJ. 2022 Feb 22;10:e12992. doi: 10.7717/peerj.12992 (PMC8877339; doi:10.7717/peerj.12992)

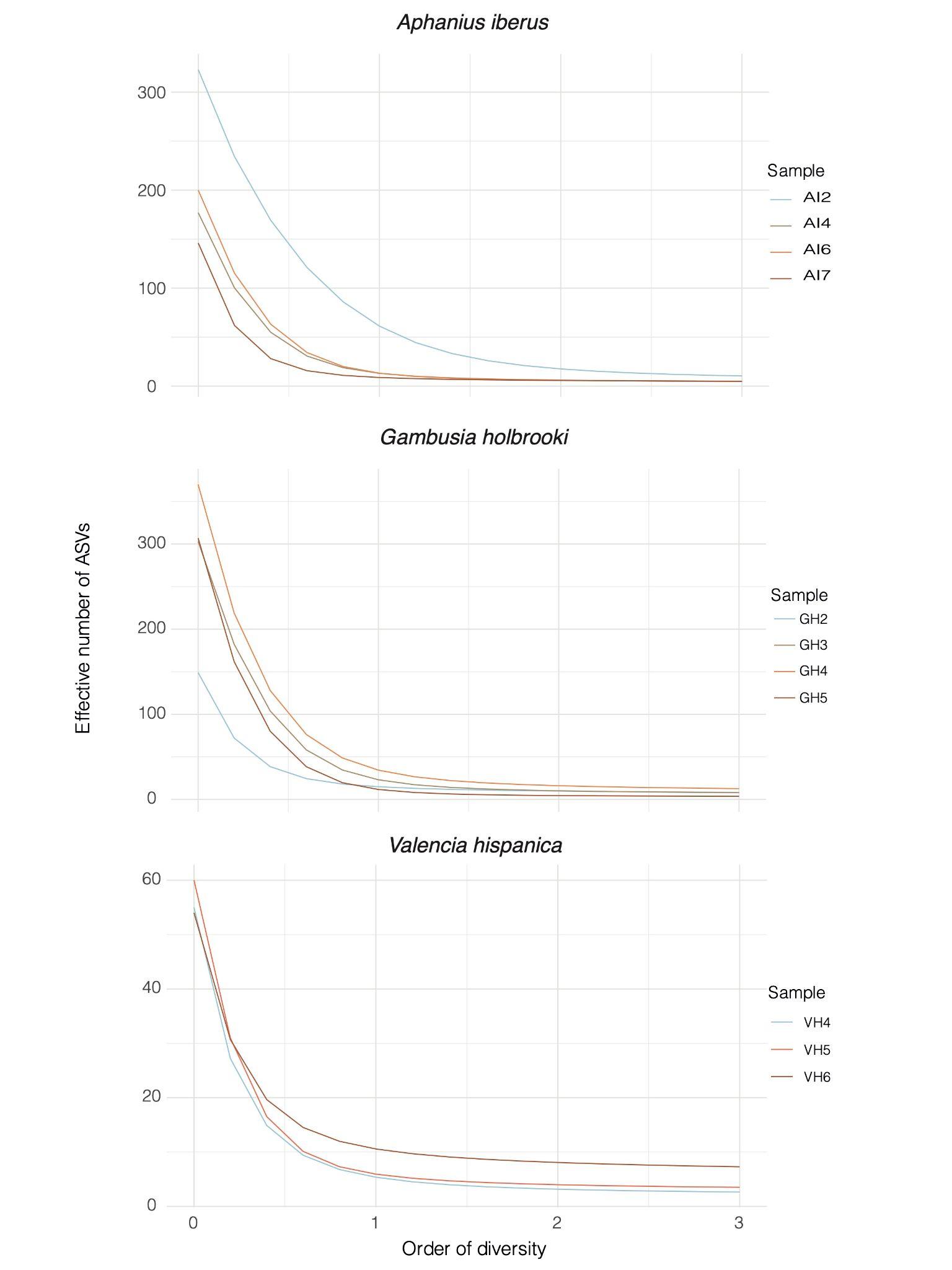

Supplement: Figure S1 — (a) Aphanius iberus, (b) Gambusia holbrooki and (c) Valencia hispanica going from q = 0 to q = 3. The higher the q-value the more weight is added to abundant ASVs. For all three fish species we observe a large drop in the effective number of ASVs going from q = 0 to q = 1 indicating that a few ASVs are dominating the communities. [file peerj-10-12992-s005.docx]

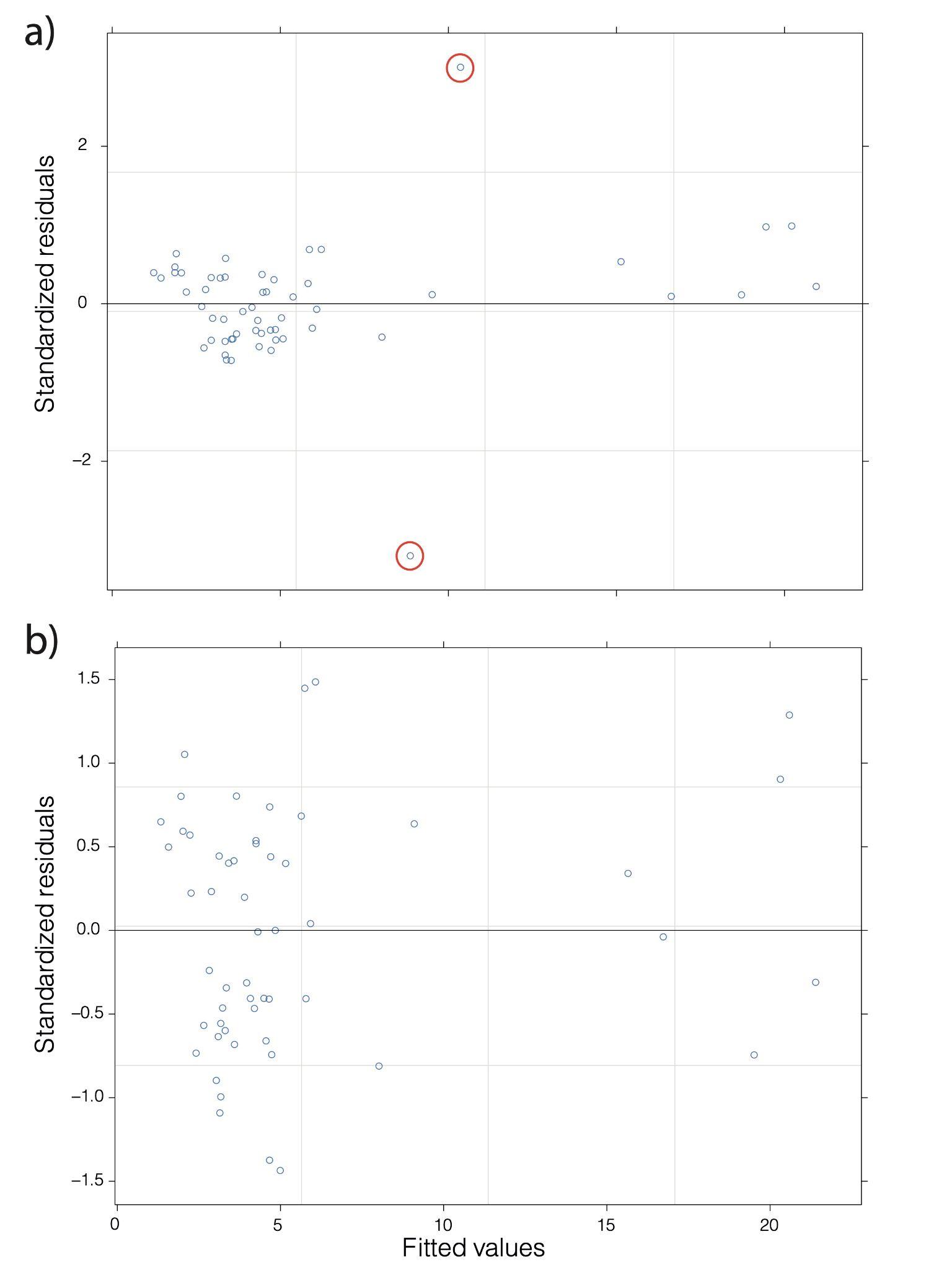
.

Supplement: Figure S2 — (a) Plots of standardized residuals vs. fitted values including outliers and (b) after moving outliers. Removing the outliers results in a more uniform distribution around the horizontal 0 line. [file peerj-10-12992-s006.docx]

**
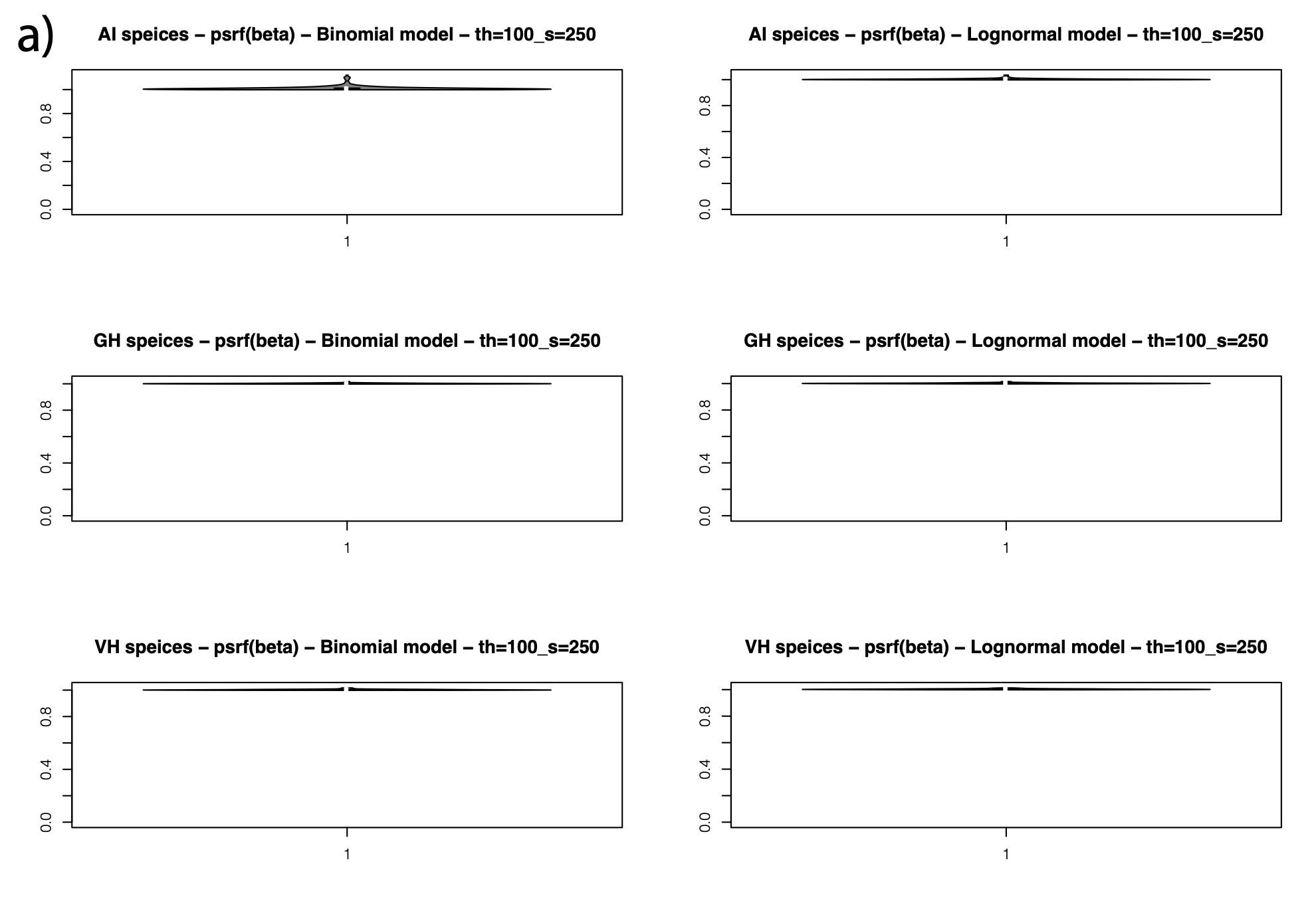
**

**
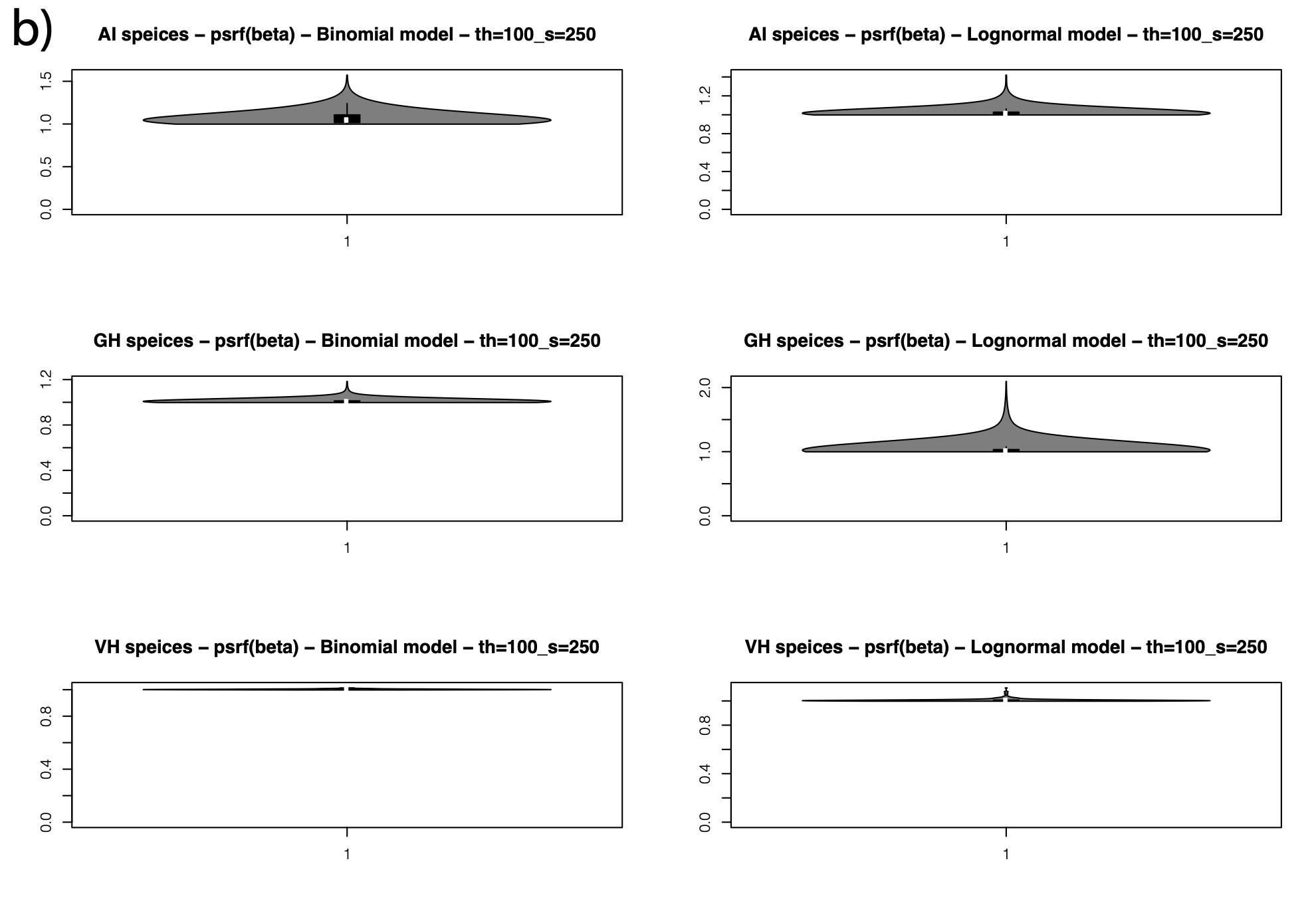
**

Supplement: Figure S3 — Convergence at (a) Family level and (b) ASV level. The plots show potential scale reduction factors for the beta parameters of the binomial (left column) and lognormal (right column) components of the models for Aphanius iberus (top), Gambusia holbrooki (middle) and Valencia hispanica (bottom). Beta parameters measure the families/ASV’s responses to explanatory variables. Potential scale reduction factors are close to 1, thus we conclude the MCMC convergence was satisfactory. [file peerj-10-12992-s007.docx]

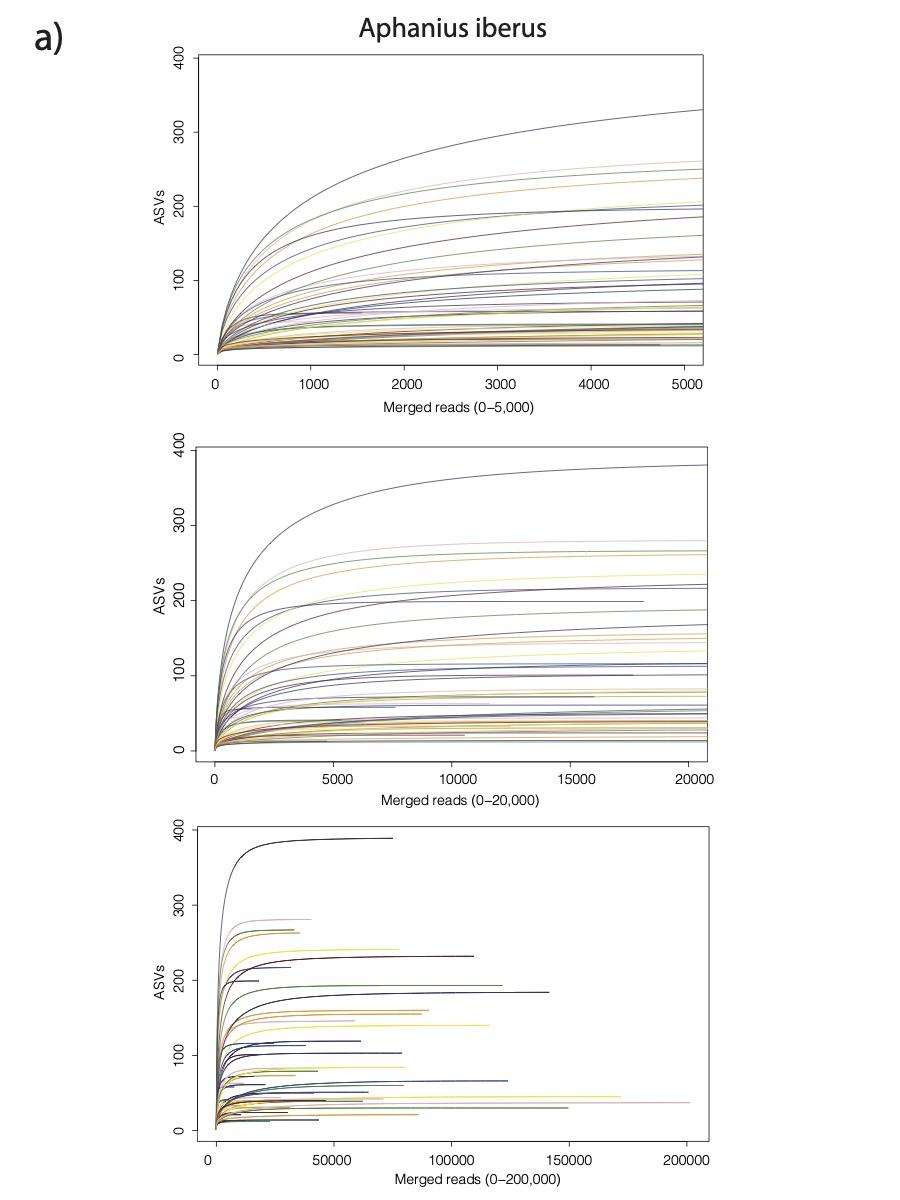


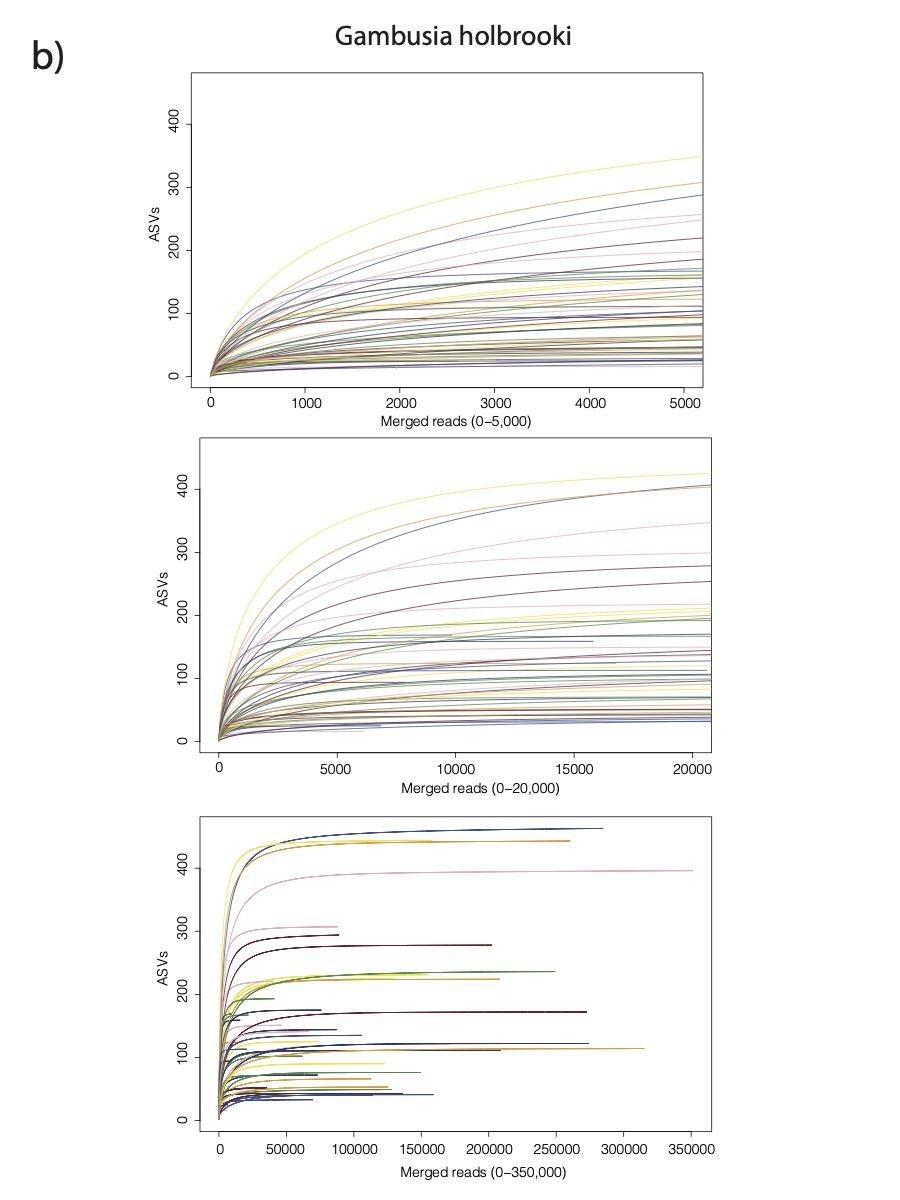


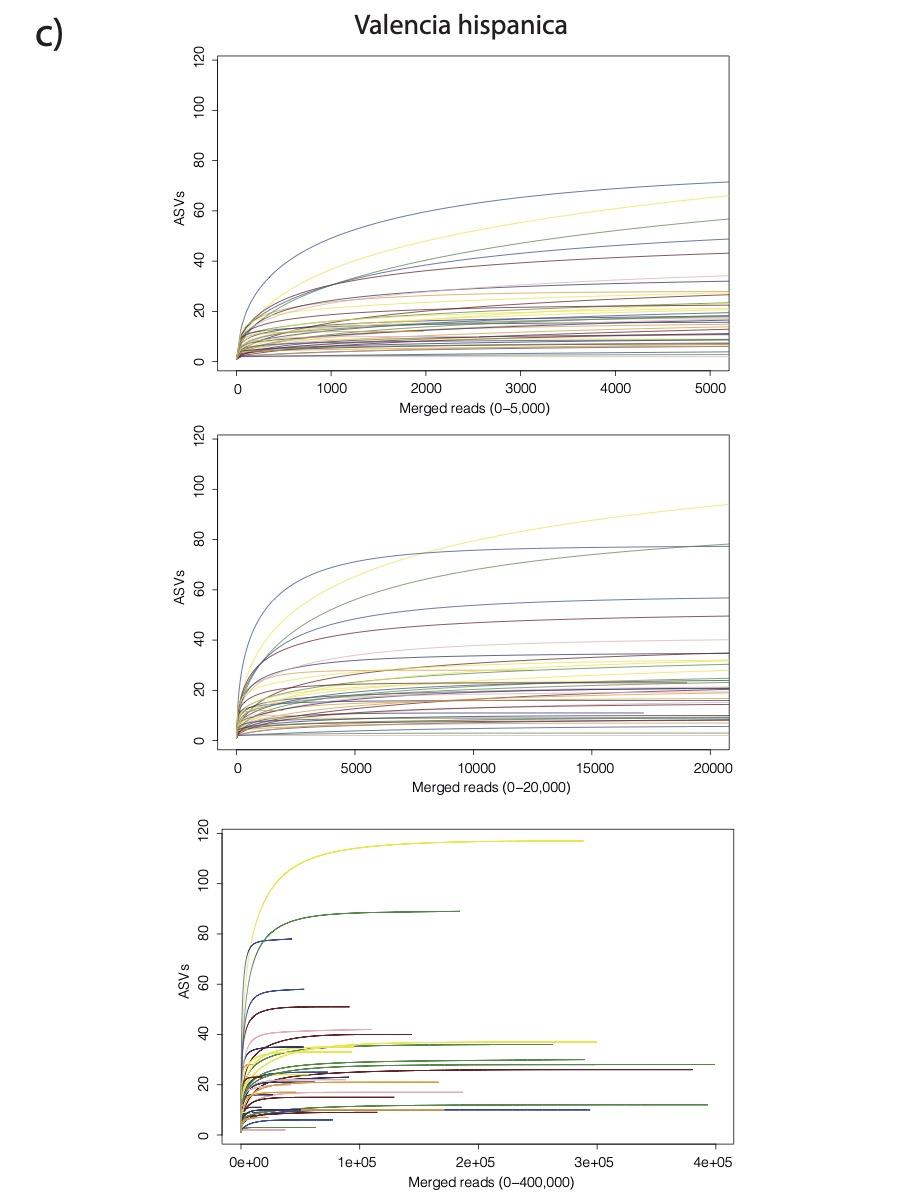

Supplement: Figure S4 — (a) Aphanius iberus, (b) Gambusia holbrooki and (c) Valencia hispanica visualizing the saturation in number of detected ASVs versus sequencing depth. [file peerj-10-12992-s008.docx]

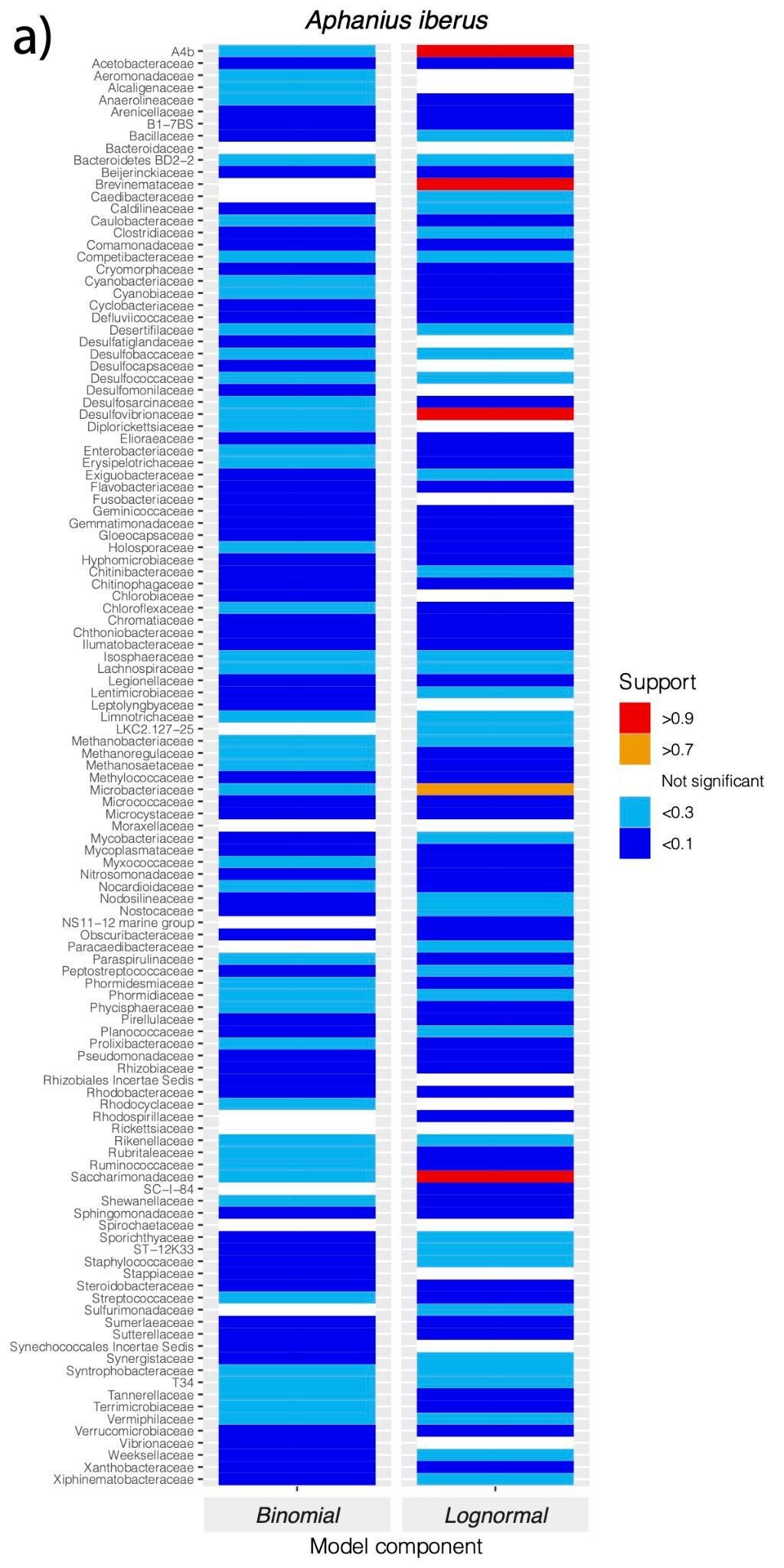


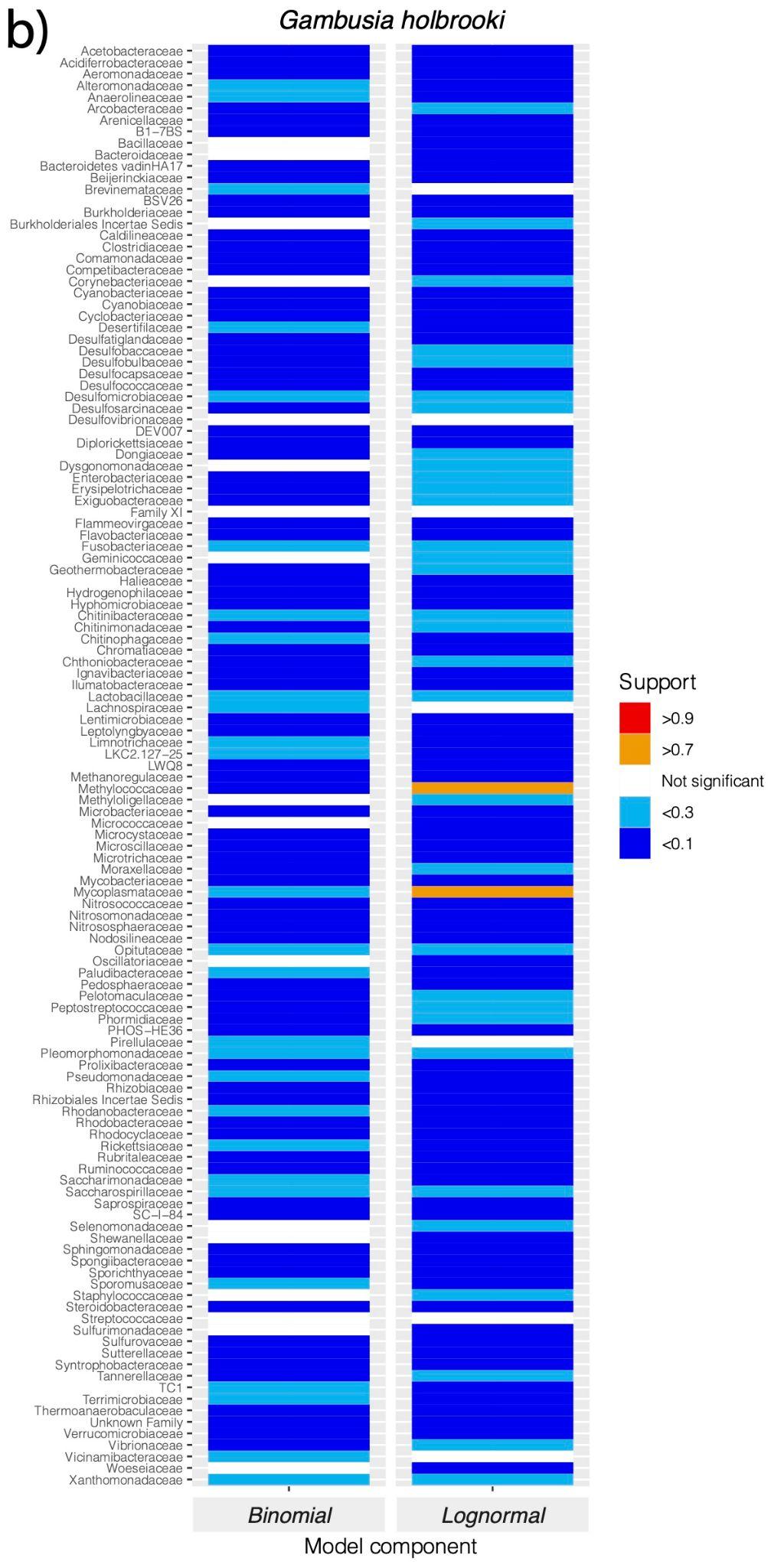


**
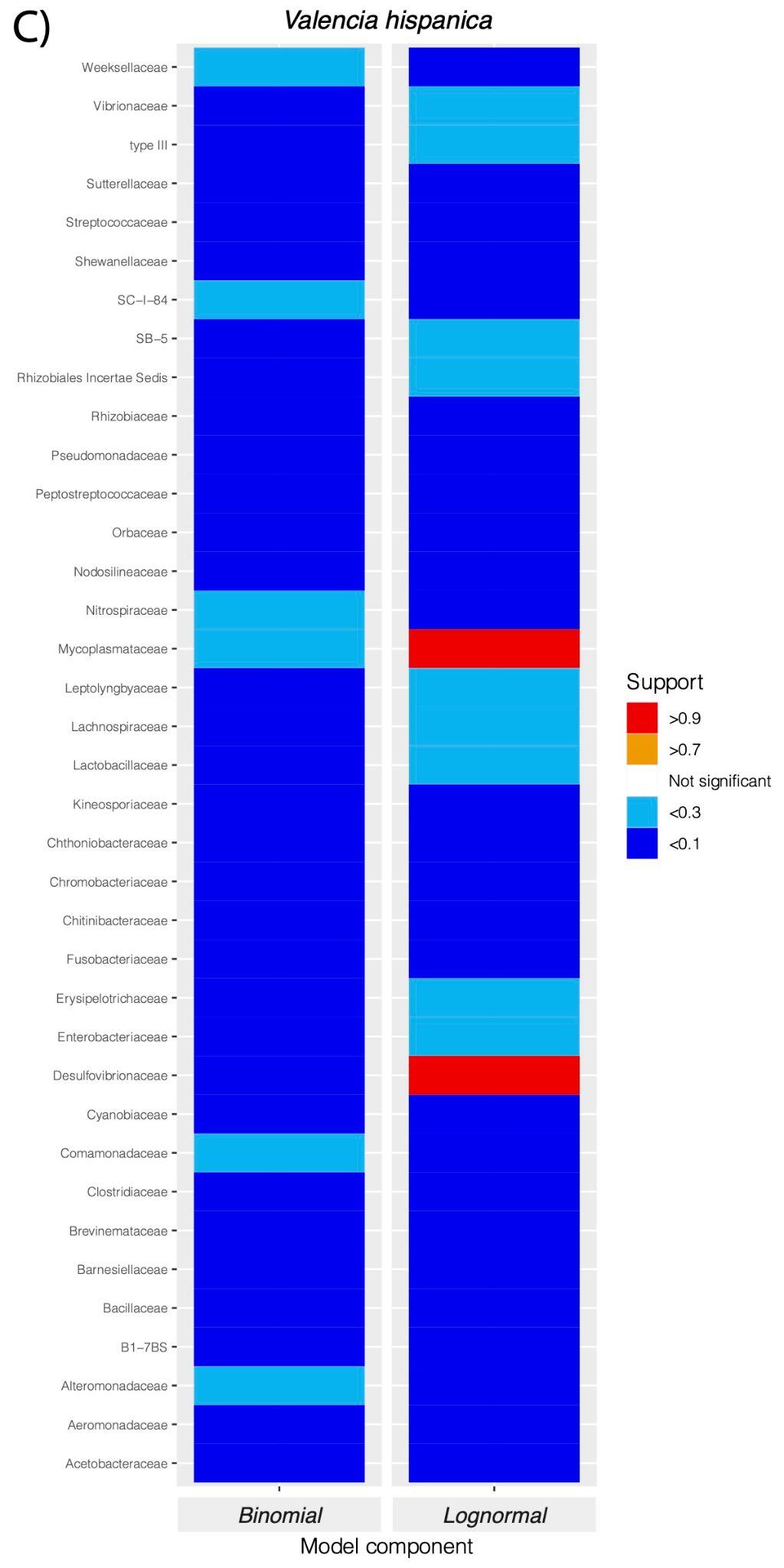
**

Supplement: Figure S5 — (a) Aphanius iberus, (b) Gambusia holbrooki and (c) Valencia hispanica. Red and orange boxes indicate positive significant associations, dark and light blue boxes indicate negative significant associations, and, white boxes indicate non-significant associations. [file peerj-10-12992-s009.docx]
